# SPARC expression is associated with hepatic injury in rodents and humans with non-alcoholic fatty liver disease

**DOI:** 10.1038/s41598-017-18981-9

**Published:** 2018-01-15

**Authors:** Guillermo Mazzolini, Catalina Atorrasagasti, Agostina Onorato, Estanislao Peixoto, Martin Schlattjan, Jan-Peter Sowa, Svenja Sydor, Guido Gerken, Ali Canbay

**Affiliations:** 1 Gene Therapy Laboratory, Instituto de Investigaciones en Medicina Traslacional, Universidad Austral, Buenos Aires, Argentina; 2 CONICET (Consejo Nacional de Investigaciones Científicas y Técnicas), Buenos Aires, Argentina; 3 Department for Gastroenterology and Hepatology, University Hospital, University Duisburg-Essen, Essen, Germany; 4 Department of Gastroenterology, Hepatology and Infectious Diseases, Otto-von-Guericke University, Magdeburg, Germany

## Abstract

Mechanisms that control progression from simple steatosis to steato-hepatitis and fibrosis in patients with non-alcoholic fatty liver disease (NAFLD) are unknown. SPARC, a secreted matricellular protein, is over-expressed in the liver under chronic injury. Contribution of SPARC accumulation to disease severity is largely unknown in NAFLD. We assessed the hypothesis that SPARC is increased in livers with more necrosis and inflammation and could be associated with more fibrosis. qrt-PCR, immunohistochemistry, and ELISA were employed to localize and quantify changes in SPARC in 62 morbidly obese patients with NAFLD/NASH and in a mouse model of diet-induced-NASH. Results were correlated with the severity of NAFLD/NASH. In obese patients 2 subgroups were identified with either high SPARC expression (n = 16) or low SPARC expression (n = 46) in the liver, with a cutoff of 1.2 fold expression. High expression of SPARC paralleled hepatocellular damage and increased mRNA expression of pro-fibrogenic factors in the liver. In line with these findings, in the NASH animal model SPARC knockout mice were protected from inflammatory injury, and showed less inflammation and fibrosis. Hepatic SPARC expression is associated with liver injury and fibrogenic processes in NAFLD. SPARC has potential as preventive or therapeutic target in NAFLD patients.

## Introduction

Obesity is a steadily growing epidemic disease that may cause a number of comorbidities including non-alcoholic fatty liver disease (NAFLD). NAFLD is currently the most common subtype of chronic liver disease in developed countries^1^, and ranges from simple steatosis (NAFL) to the more severe steatohepatitis (NASH)^2^. NASH patients may progress to cirrhosis and hepatocellular carcinoma^1^. Nearly 85–95% of morbidly obese patients have NAFLD and more than 30% of them have NASH^3^. Despite this major clinical relevance, the factors driving the progression from relatively benign steatosis to NASH remain poorly defined. Currently simple steatosis is distinguished from NASH histopathologically by the NAFLD Activity Score (NAS), including inflammation, hepatocyte ballooning, and steatosis^4^. As non-invasive methods to detect NAFLD severity various factors have been tested including markers of cell death, pro-fibrogenic factors, and adipokines^2,5,6^. Though, none of these factors have been widely accepted for clinical routine diagnostics, yet^7^.

Secreted protein, acidic and rich in cysteine (SPARC), also named osteonectin or BM-40, is a secreted multifunctional extracellular matrix (ECM)-associated protein. SPARC is involved in different biological processes including wound healing response to injury, tissue remodeling and fibrosis^8,9^. We and others have observed SPARC over-expression in activated hepatic stellate (HSC) and liver sinusoidal endothelial cells (LSEC) in cirrhotic livers from mice and patients^10–14^. SPARC was found to stimulate collagen deposition, inflammation, TGF-β1 production, and ECM proteins synthesis^13,14^. Due to this connection to fibrogenic processes SPARC was proposed as a therapeutic target to prevent fibrosis in chronic inflammatory and profibrogenic conditions^15^.

SPARC is also present in adipose tissue, and its expression and secretion are partially regulated by insulin and glucose levels^16^. SPARC is involved in adipogenesis^17^ and SPARC-null mice exhibit an increased adipose tissue accumulation^18^. In addition, in patients with gestational diabetes mellitus and obesity SPARC levels correlated with dyslipidemia and insulin resistance^19–21^. Therefore, it seems that SPARC may contribute to metabolic dysregulation in obesity.

However, the role of SPARC in NAFLD/NASH patients has not been explored, yet. In the present project we investigated if SPARC is connected to severity of liver injury in NAFLD. In particular we tested the hypothesis that SPARC is elevated under conditions of necrosis and inflammation, and could be associated with a high risk of fibrosis in obesity-related human liver disease and animal models mimicking NAFLD/NASH. We observed low SPARC expression was associated with a protection from NASH in a mouse model and reduced liver injury in morbidly obese patients.

## Experimental Procedures

### Patients

The study population consisted of 62 Caucasian patients undergoing bariatric surgery (BAS) at the Department II of Surgery, Alfried-Krupp Hospital Essen, Germany. All patients met the following criteria for surgical weight loss therapy established by the NIH consensus conference in 1991^22^: age >18 years, severe obesity with a BMI (body mass index) ≥40 or ≥35 kg/m with co-morbidities, failure of medical weight loss, absence of medical or psychological contra-indications for BAS, and evaluation by a multi-disciplinary team of medical, nutrition, psychiatry and surgical specialists. Demographic and clinical data included age, gender, BMI, liver enzymes and metabolic parameters. Patients aged <18 or >65 years with liver pathologies other than NAFLD, history of organ transplantation, history of malignancy within the previous 5 years, alcohol abuse defined as an average daily consumption of >20 g/day for women and >30 g/day for men, drug abuse within the previous year, autoimmunity or genetic disorders, and therapy with immunosuppressive or hepatotoxic agents were excluded. A total of ten subjects with normal BMI (>18.5 and <25) without any signs of liver disease were used as healthy controls.

### Surgical intervention

BAS was carried out by laparoscopic approach in all patients. Operations were either performed as Roux-en-Y gastric bypass, sleeve gastrectomy or gastric banding according to surgeons’ choice. All patients were informed about the additional risks of a wedge liver biopsy during the bariatric procedure. Liver specimens were split and stored in either 4% (v/v) formalin solution (Roth) for subsequent histological examination or in RNAlater (Ambion Applied Biosystems) for RNA isolation. The study was conducted in accordance with the ethical guidelines of the 2008 Helsinki Declaration and the ethics committee of the University Hospital of Essen approved the protocol (09–4252). All patients provided written informed consent before enrolment.

### Animal Studies

*In vivo* experiments were performed in male C57BL/6J mice with 20 to 25 g of body weight. SPARC knockout mice (SPARC^−/−^) on a C57BL/6 background were purchased from Jackson Laboratory, USA. Animals were maintained in a temperature (24 °C) and light controlled (12:12 h light:dark) facility, and had free access to food and water. At 8 weeks of age SPARC^−/−^ and SPARC^+/+^ mice were randomized to 4 treatment groups (6–8 mice per group): group 1) SPARC^+/+^ fed *al libitum* with high fat diet and 50 g/L sucrose was added to drinking water (termed Western diet, WD); group 2) SPARC^+/+^ fed *ad libitum* with regular laboratory chow (RD); group 3) SPARC^−/−^ fed *al libitum* with WD; group 4) SPARC^−/−^ fed *al libitum* with RD^23,24^. All mice were sacrificed after 20 weeks on the WD or RD diets. The WD contains calcium caseinate (200 g/kg), vitamin mixture (10 g/kg), cellulose (50 g/kg), animal fat (250 g/kg), vitamin A (1 ml/kg), choline bitartrate (2.5 g/kg), maltodextrin (451.5 g/kg). The mixture of vitamins and minerals are prepared according to the recommendations of the AIN 93^25^. Animals in each group were euthanized after 20 weeks. All protocols dealing with animals were reviewed and approved by the Austral University Animal Studies Committee. This study followed the guidelines outlined in the National Institutes of Health Guide for the Care and Use of Laboratory Animals.

### qrtPCR (quantitative real-time PCR)

Murine liver tissue was homogenized and total RNA was extracted by Trizol Reagent (Sigma-Aldrich Co., USA). RNA (1 μg) was reverse transcribed with 200 U of SuperScript II Reverse Transcriptase (Invitrogen, USA) using 500 ng of Oligo (dT) primers. cDNAs were subjected to qPCR. SPARC, IL6, α-SMA, IP10, collagen type I, Fas, and TNFα mRNA levels were quantified by SYBR® Green (Invitrogen) qPCR (Stratagene Mx3005p, Stratagene, USA). All PCR amplifications were carried out using 40 cycles of 95 °C for 30 s, 55 °C for 30 s, and 72 °C for 30 s. mRNA levels for GAPDH were used as the endogenous controls. Tissue from human liver biopsies was homogenized with a blade homogenizer. Total RNA was isolated with the RNeasy mini kit (Qiagen, Hilden, Germany) following manufacturers’ instructions. PCR of cDNA was performed using the iCycler iQ thermal cycler (Bio-Rad Laboratories, Munich, Germany). Relative gene expressions were calculated from the threshold cycles in relation to the reference gene HPRT (hypoxanthine-guanine phosphoribosyltransferase) in healthy controls. Changes in gene expression were expressed as 2^ΔΔCt^ referred to as fold change compared to the mean expression quantified in healthy livers. See supplementary table for list of oligonucleotides utilized for qrtPCR.

### Histopathology

Liver tissue was fixed in 10% Formalin and embedded in paraffin. Hematoxylin and eosin liver specimens were evaluated by light microscopy. An experienced pathologist assessed steatosis, inflammation, and ballooning in a blinded fashion. Steatosis, inflammation, and ballooning were scored based on the NAS^4^. For liver fibrosis assessment liver sections were stained with picrosirius red staining, and collagen fibers were quantified as described by Camino *et al*.^13^. Fresh liver tissue was frozen in optimal cutting temperature compound and stained with Oil Red O for lipid analysis^26^.

### Immunostaining

Human tissue sections were pre-incubated with Block-ace (Dako Cytomotion) for 10 min at 37 °C to block nonspecific binding of the primary antibody. Endogenous peroxidase activity was blocked with 0.3% H_2_O_2_ and 0.1% sodium nitrite in distilled water for 10 min at room temperature (20 °C). Sections were incubated overnight with 1:150-diluted SPARC-specific monoclonal antibody, rinsed three times with PBS and incubated with avidin–biotin peroxidase complexes (Vector Laboratories, Burlingame, USA). Slides were developed with a DAB (3,3′-diaminobenzidine) substrate kit (Vector Laboratories, Burlingame, USA). Sections were counterstained for 3 min with haematoxylin and cover-slipped with mounting medium for light microscopy.

Immunohistochemistry staining of mice specimens was performed according to the manufacturer’s instructions. Briefly, antigen retrieval was carried out in a pressure cooker by boiling in 10 mM citrate buffer (pH 6.0), followed by washing with phosphate-buffered saline. Subsequently, endogenous peroxidase was quenched with 3% H_2_O_2_ for 10 min at room temperature. After rinsing, the slides were treated overnight at 4 °C with a negative control reagent or α-smooth muscle actin (αSMA; rabbit polyclonal; 1:200; Abcam). Slides were incubated with anti-rabbit horseradish peroxidase-conjugated secondary antibody (Vector Laboratories, Burlingame, USA). Binding to the antibody was detected using the labeled polymer method. Diaminobenzidine was used as the chromogen. Perisinusoidal positive cells were quantified using Image J, large central and portal areas were excluded. Twenty random fields (400× magnification) were analyzed for each group. For F4/80 immunofluorescence, frozen liver sections were stained using an anti-F4/80 (1:250, Abcam, USA) primary antibody and an anti-rat Cy3-conjugated IgG secondary antibody (1:400, Jackson, USA). For quantitative assessment of protein expression, 10 randomly selected fields at 200× magnification were captured for each specimen and quantified using ImageJ software (US National Institutes of Health, Bethesda, MD, USA).

### ELISA

Adiponectin and SPARC concentrations (both as Quantikine ELISA® kits; R&D Systems) were determined by sandwich ELISA. Plates pre-coated with human monoclonal antibodies were blocked by adding 15% (w/v) BSA, washed and incubated with sera from the patients.

The cell death markers M30 (for apoptosis) and M65 (total cytokeratin-18/overall cell death) were assessed in patients’ sera using the M30 (Apoptosense) and M65 ELISA® kits (both from Tecomedical, Sissach, Switzerland) following the manufacturer’s instructions. Absorbance for all ELISA kits was measured at 450 nm.

### Statistical Analysis

Data are reported as arithmetic means ± SEM. All data were analyzed using Prism version 6 software (GraphPad, Carlsbad, USA). Statistical significance was determined with the appropriate test depending on data distribution and number of groups. Two-way ANOVA with Bonferroni correction for multiple comparison, one-way ANOVA Kruskal-Wallis test with Dunn’s multiple comparisons post-test, or the Mann–Whitney U-test (parameters in human samples were not normally distributed) were applied, respectively. For all analyses, a P-value < 0.05 was considered statistically significant. For correlation analysis Spearman rank correlation was applied.

## Results

### SPARC is expressed in patients with NAFLD

SPARC is expressed in mice and patients with cirrhosis^10,11,14^, but no studies have been performed in NAFLD, yet. To address this point, we analyzed liver biopsies and serum from 62 patients with morbid obesity undergoing BAS. The principal characteristics of the patient population are shown in Table 1.

**Table 1:** Demographic and clinical data of obese patients separated by hepatic SPARC mRNA expression.

	SPARC low (n = 46)	SPARC high (n = 16)
Age at surgery (y)	50.7 ± 1.5	47.6 ± 2.3
Sex (m/f)	10/36	2/14
BMI (kg/m²)	51.0 ± 1.2	51.5 ± 1.7
Fasting glucose (mg/dl)	99.9 ± 4.5	115.0 ± 13.5
HbA1c (%)	5.7 ± 0.1	6.0 ± 0.2
Leukocytes (/nl)	14. ± 6.1	8.4 ± 0.5
Thrombocytes (/nl)	266.5 ± 12.5	300.2 ± 15.9
Erythrocytes (10^6^/ml)	5.5 ± 0.8	4.8 ± 0.1
Bilirubin (mg/dl)	0.55 ± 0.05	0.50 ± 0.06
LDH (IU/l)	230.5 ± 8.3	213.3 ± 14.5
Creatinine (mg/dl)	0.98 ± 0.13	0.76 ± 0.05
Total cholesterol	197.6 ± 6.1	196.4 ± 6.2
LDL (mg/dl)	135.1 ± 4.8	127. ± 5.0
HDL(mg/dl)	44.8 ± 1.6	46.5 ± 2.5
Adipocyte cell diameter (µm)^1^	125 (75–150)	125 (100–150)
Adiponectin (ng/ml)	3.6 ± 0.4	4.1 ± 0.5
Steatosis^1^	1 (0–3)	1 (0–3)
Inflammation^1^	1 (0–3)	1 (1–3)
Ballooning^1^	1 (0–2)	1 (0–2)
NAFLD activity score^1^	3 (1–6)	3 (1–6)

Data is given as mean ± standard error of mean. ^1^Data for these parameter is given as mean (range).

**Figure 1:**
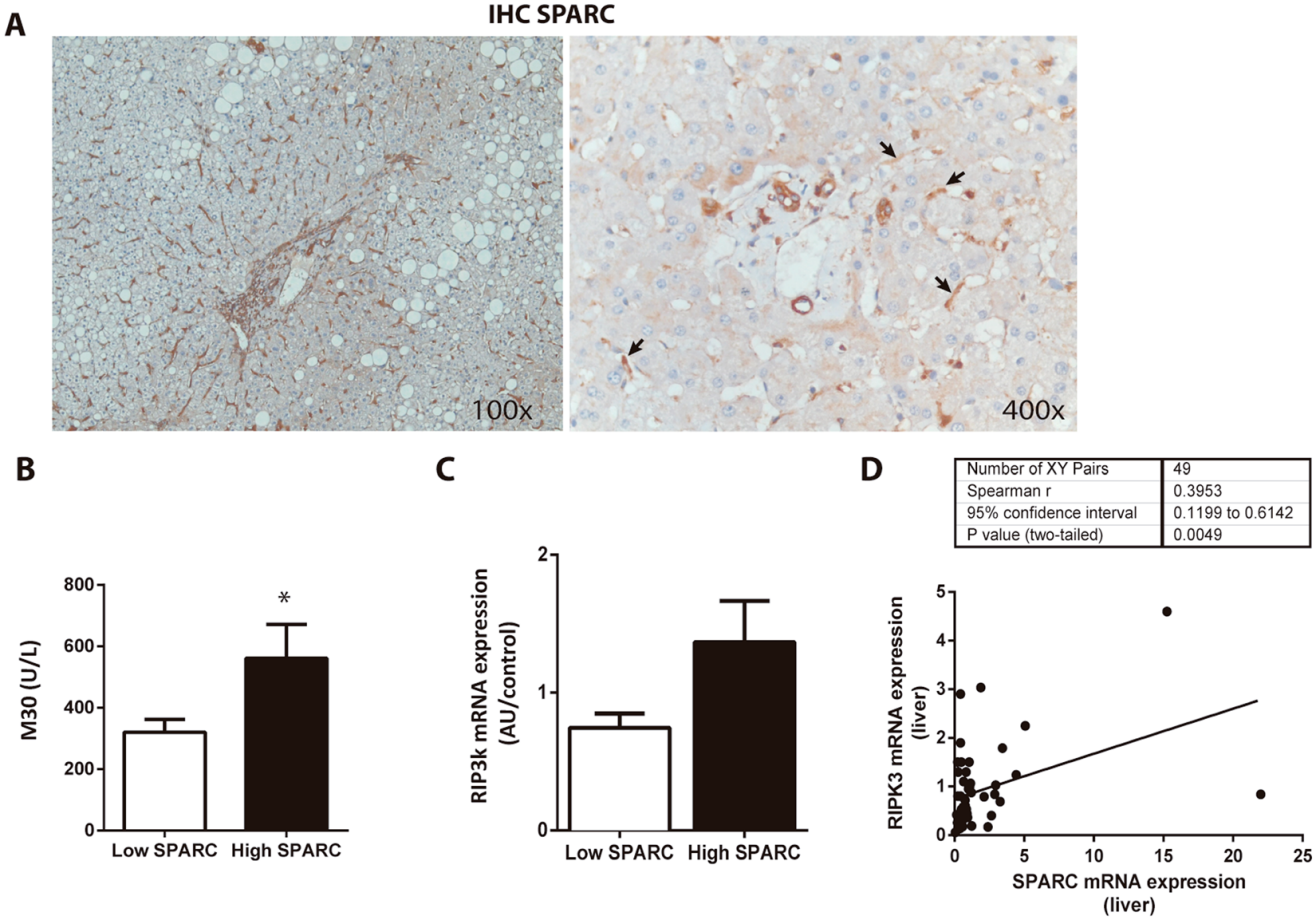
SPARC expression in patients with NAFLD induced liver injury. (**A**) Expression of SPARC was observed on protein (immunohistochemistry) in patients with NAFLD. Magnification 100× and 400×. Patients with high hepatic SPARC mRNA expression had significantly higher serum M30 levels (**B**) and nominally higher RIPK3 mRNA expression (**C**). Data are mean ± sem, *p < 0.05. Comparison between groups was performed with Mann-Whitney U Test. mRNA expressions of SPARC and RIPK3 were significantly correlated (**D**). Spearman rank test.

SPARC expression was detected in liver tissue of NAFLD patients by qPCR and IHC (Fig. 1A). SPARC staining localized mainly within non-parenchymal cells. However, some positive staining was also observed within hepatocytes. SPARC expression values in liver tissue from the 62 patients ranged from a fold change of 0.05 to 21.9 of the expression in healthy controls. SPARC expression was increased (values greater than 1.2 fold change) in 25.8% of patient. Thus, patients were grouped by either high or low SPARC mRNA expression (high SPARC group, n = 16; low SPARC group, n = 46) at a cut-off of 1.2 fold change. Serum protein content of SPARC did not differ between the groups.

### Markers of liver injury increase with NAFLD-related expression of SPARC in humans

Routine serum markers of hepatocyte injury did not differ significantly between patients with low or high SPARC expression in the liver and remained well within normal ranges. Although serum AST and ALT concentrations were nominally higher in the SPARC high group compared to low hepatic SPARC expression, this difference was not statistically significant (AST: 27.3 ± 1.5 vs 37.5 ± 5.5, p = 0.06; ALT: 34.6 ± 3.2 vs 46.3 ± 7.9, p = 0.24; low SPARC vs high SPARC group). We also analyzed serum concentrations of cytokeratin-18 (M65), as surrogate marker for overall cell death, and caspase-cleaved cytokeratin-18 (M30), as surrogate marker of apoptosis. The SPARC high group exhibited higher concentrations of M30 (p = 0.019; Fig. 1B). For M65 no difference was observed between the groups. Despite higher markers of epithelial apoptosis no differences in histological inflammation were observed between the SPARC groups (Table 1). Receptor interacting protein-1 (RIP1) and −3 regulate necroptosis^27^ and might be involved in development of NASH^28^. Patients with high levels of SPARC exhibited nominally higher expression of hepatic RIP3k assessed by qPCR (p = 0.084; Fig. 1C). In addition, we found a positive correlation between SPARC and RIP3k mRNA expression in the liver (Spearman = 0.3953; p = 0.005; Fig. 1D). Hepatic apoptosis and possibly necroptosis could be associated with higher SPARC mRNA expression in NAFLD.

### Fibrosis markers correlate with SPARC expression in patients with NAFLD

**Figure 2:**
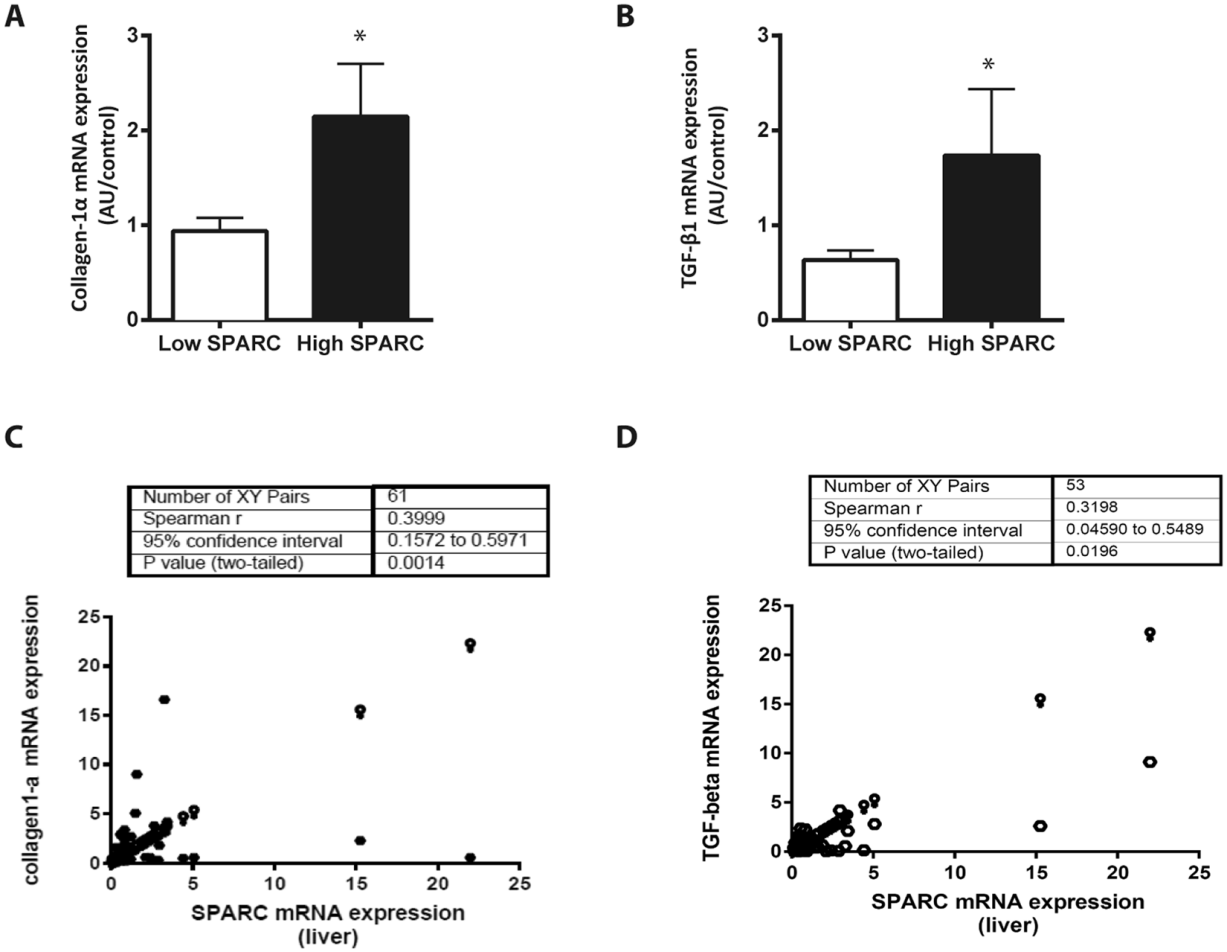
Pro-fibrogenic gene expression is increased in patients with NAFLD and high SPARC expression. NAFLD patients were grouped by hepatic SPARC mRNA expression. Patients with high SPARC expression had significantly higher collagen 1 alpha (**A**) and TGF-beta (**B**) mRNA expression in the liver. Data are mean ± sem, *p < 0.05. Comparison between groups was performed with Mann-Whitney U Test. mRNA expressions of both pro-fibronic genes were significantly correlated to SPARC expression (**C**,**D**). Spearman rank Test.

As described above SPARC is connected to fibrogenesis^13^ and cirrhosis^10,11,14^. To confirm this association in NAFLD, we checked markers of fibrosis. Indeed, patients with high SPARC expression showed significantly higher mRNA levels of collagen Iα (Fig. 2A) and TGF-β1 mRNA expression (Fig. 2B), as observed in other etiologies^10,11,13^. Spearman correlation analysis resulted in positive correlations between SPARC mRNA expression and both collagen Iα (Fig. 2C) as well as TGF-β1 (Fig. 2D) expressions in the liver. Though, expression of a-SMA mRNA, a marker for HSC activation, and collagen deposition assessed by Sirius red staining were not significantly different between the two groups (data not shown). In NAFLD patients hepatic SPARC expression seems to be associated with early genes of fibrogenesis.

### SPARC and adipose tissue

**Figure 3:**
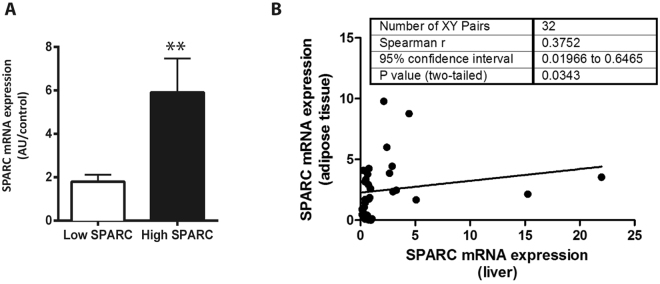
SPARC expression in adipose tissue and liver tissue are correlated. NAFLD patients were grouped by hepatic SPARC mRNA expression. Patients with high hepatic SPARC expression also had significantly higher SPARC mRNA expression in adipose tissue (**A**). Data are mean ± sem, **p < 0.01, Mann-Whitney U Test. mRNA expressions of SPARC in both sites were significantly correlated to each other (**B**). Spearman rank test.

SPARC expression has been found elevated in subcutaneous and visceral adipose tissue from obese patients^20^. It is also known that SPARC inhibits adipogenesis by differentiation of preadipocytes into adipocytes and SPARC knockout aggravates diet induced obesity^17^. We have previously observed that there is a crosstalk between adipose tissue and the liver^29^, which was corroborated by an association between adipose tissue inflammation and histological severity of NAFLD^30^. Thus, we tested if SPARC expression could mediate this effect. SPARC mRNA expression in visceral adipose tissue was significantly higher in patients of the high SPARC group (Fig. 3A). As a result we observed a positive correlation between hepatic and adipose tissue expression of SPARC (Spearman = 0.3752; p = 0.03; Fig. 3B). SPARC seems to be co-regulated in adipose and liver tissue in NAFLD patients.

### SPARC and NASH mouse model

**Figure 4:**
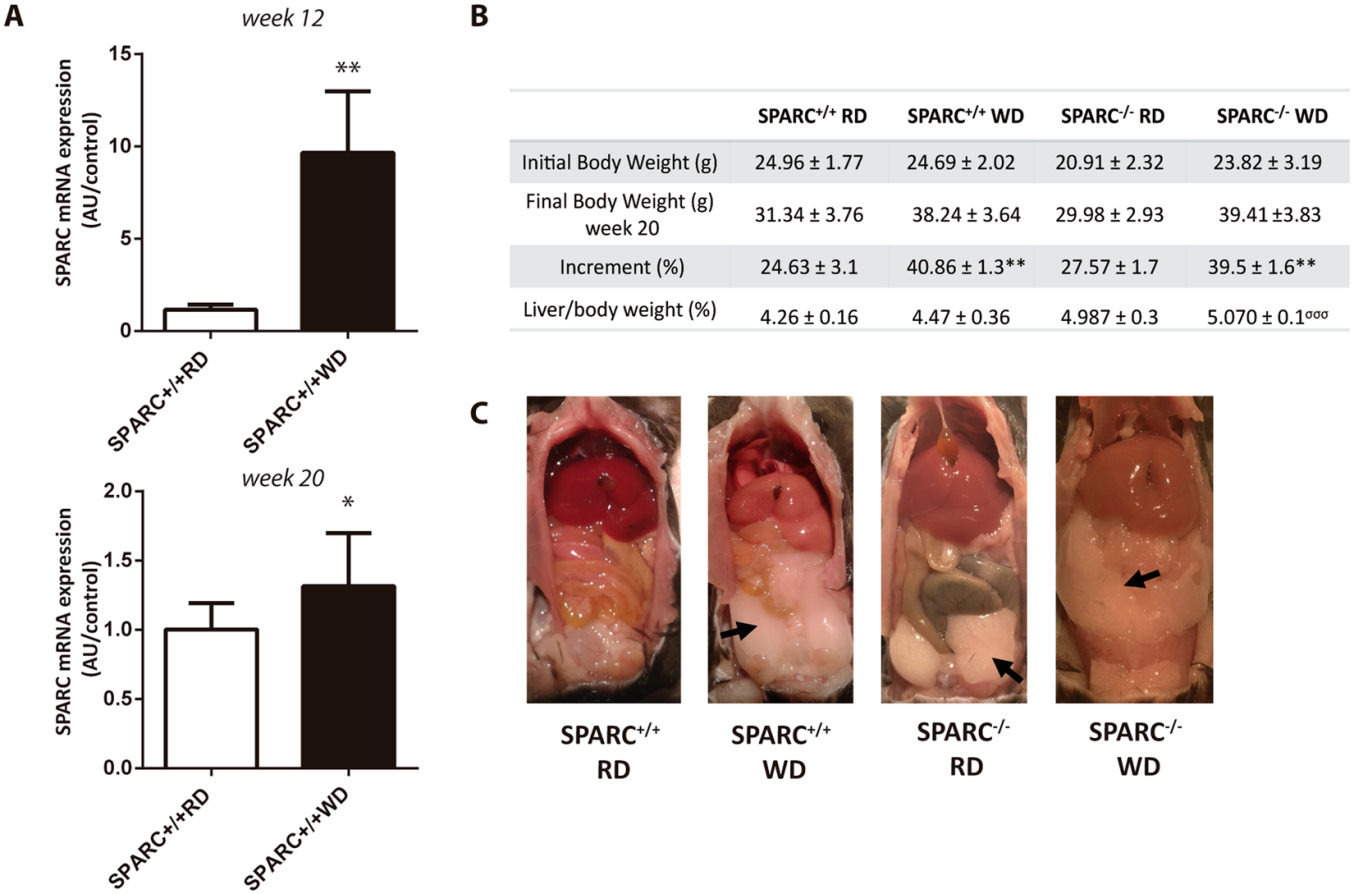
SPARC expression in murine dietary NASH model. (**A**) Hepatic SPARC expression detected by qPCR in the high fat diet (HF) model of NASH in mice. After 12 (upper panel) and 20 (lower panel) weeks of western diet (WD), SPARC expression was significantly induced compared with mice fed with regular chow (RD). (**B**) Body and liver weight increment after 20 weeks. Data are means ± SEM. *, compared between WD and RD diet; ^σ^, compared between SPARC^−/−^ and SPARC^+/+^ fed with WD diet. *p < 0.05, **p < 0.01, ^σσσ^p < 0.001; Kruskal-Wallis test. (**C**) Visceral adipose tissue deposition (black arrow) and appearance of the liver in SPARC^−/−^ and SPARC^+/+^ fed with WD or RD diet for 20 weeks.

**Figure 5:**
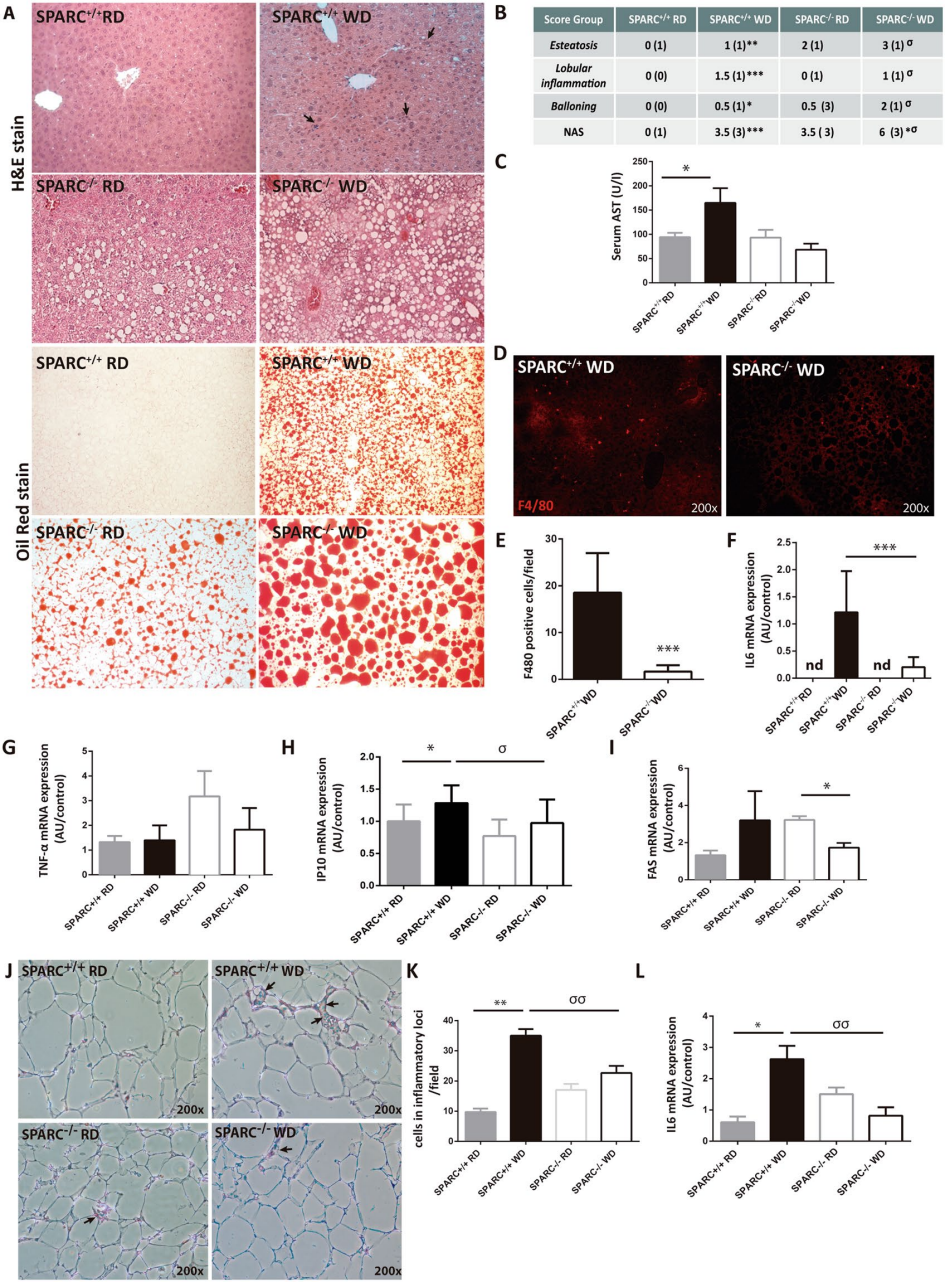
Effect of SPARC deficiency in mice fed with a non-alcoholic steatohepatitis-inducing diet. (**A**) Hematoxylin/eosin (H/E) and oil red staining of liver sections from SPARC^+/+^ mice and SPARC^−/−^ mice after feeding with WD or RD diet for 20 weeks. H/E staining demonstrated more inflammation and necrosis in WD-fed SPARC^+/+^ mice compare with WD-fed SPARC^−/−^ mice. Arrows indicate inflammatory cell infiltration. WD diet increased fat deposition visualized by oil red staining in SPARC^+/+^ and SPARC^−/−^mice. WD-fed SPARC^−/−^ exhibited enlarged lipid droplets compared to SPARC^+/+^ with WD. Image magnification: 200×. (**B**) NAFLD activity score (NAS) showing more steatosis, inflammation and ballooning in SPARC^+/+^ mice in comparison with SPARC^−/−^ mice. n = 6–8 mice per group. Data are presented as median and range. *Compared WD vs RD diet in both SPARC^+/+^ and SPARC^−/−^ mice; ^σ^compared WD-fed SPARC^+/+^ versus WD-fed SPARC^−/−^ mice. *p < 0.05, ***p < 0.001; ^σ^p < 0.05; ^σσ^p < 0.01, ^σσσ^p < 0.001; Kruskal-Wallis test with Dunn’s post test C) Serum AST levels. Data are mean ± sem. *p < 0.05, Kruskal-Wallis test with Dunn’s post test. n = 6–8 mice per group. D) Analysis of hepatic F4/80 positive cell infiltration by immunofluorescence in liver sections of WD diet-fed SPARC^+/+^ and SPARC^−/−^ mice. Magnification: 200× (**E**) F4/80 positive cells quantification. Ten random fields were analyzed for each group (x200 magnification), n = 4 mice per group. ***p < 0.001, Mann-Whitney T test. (**F**) Hepatic IL6 mRNA expression assessed by qrt-PCR ***p < 0.001, Mann-Whitney T test. nd, non-detectable amplification (**G**–**I**) Hepatic mRNA expression of tumor necrosis factor-α (TNF-α), IP-10, and FAS/CD95 in response to WD diet in both SPARC^−/−^ mice and SPARC^+/+^ mice compared with animals fed with RD. (**J**) Hematoxylin/eosin (H/E) staining of adipose tissue sections from SPARC^+/+^ and SPARC^−/−^ mice after feeding with WD or RD diet for 20 weeks. Magnification: x200. (**K**) Inflammatory cell quantification. Ten random fields were analyzed for each group (x200 magnification). (**L**) Adipose IL6 mRNA expression assessed by qPCR. *Compared WD vs RD diet in both SPARC^+/+^ and SPARC^−/−^ mice; ^σ^compared WD-fed SPARC^+/+^ versus WD-fed SPARC^−/−^ mice. * or ^σ^p < 0.05, ** or ^σσ^p < 0.01, Kruskall-Wallis test, with Dunn’s post test.

Hepatic SPARC expression was analyzed in a WD model of NASH in mice. After 12 and 20 weeks of WD, SPARC expression was significantly induced compared with mice fed RD (Fig. 4A). To get insight into the function of enhanced SPARC expression in NAFLD, we compared SPARC-deficient (SPARC^−/−^) mice and wild type (SPARC^+/+^) mice in a well–established dietary NASH model fed for 20 weeks^31,32^. In accordance with previous observations adipose tissue depots were more pronounced in SPARC^−/−^ mice (Fig. 4C)^18^. Although SPARC^−/−^ mice had larger visceral adipose tissue depots, the overall body weight of WD-fed mice after 20 weeks was not significantly different to *wt* mice. This is in agreement with previous data^8^ (Fig. 4B). Liver/body weight ratio was significantly higher in SPARC^−/−^ mice fed with WD compared with SPARC^+/+^ mice (Fig. 4B). In line with this, WD caused higher macrovesicular steatosis in SPARC^−/−^ mice in comparison with SPARC^+/+^ mice, demonstrated by HE and Oil Red O staining (Fig. 5A). The amount of steatosis was significantly higher in SPARC^−/−^ mice; however, serum AST levels were higher in WD-fed SPARC^+/+^ mice in comparison with WD-fed SPARC^−/−^ mice at week 20 (Fig. 5C). SPARC^+/+^ mice showed more histological inflammation than SPARC^−/−^ mice (Fig. 5A,B) quantified by NAFLD activity score (NAS). SPARC^−/−^ mice had a median score of 1 while SPARC^+/+^ mice had a median score of 1.5, respectively. In agreement with this observation, SPARC^+/+^ mice showed higher hepatic expression of pro-inflammatory cytokines and chemokines in response to WD compared with SPARC^−/−^ mice. Expression levels of IL-6, IP-10 (CXCL10), and FAS/CD95 were increased (Fig. 5F–I). The type of hepatic mononuclear inflammatory infiltration was characterized by immunofluorescence, revealing an increased number of F4/80 positive cells in SPARC^+/+^ mice when compared with SPARC^−/−^ mice (Fig. 5D,E). This data suggests that the absence of SPARC is associated with a lower degree of hepatic Kupffer cell/macrophage activation. We studied abdominal adipose tissue and observed that SPARC^−/−^ mice fed with WD showed less inflammatory cell infiltration and decreased IL6 mRNA expression in adipose tissue in comparison with SPARC^+/+^ mice fed with WD (Fig. 5J–L).

### Fibrosis is decreased in SPARC^−/−^ mice fed with Western diet

**Figure 6:**
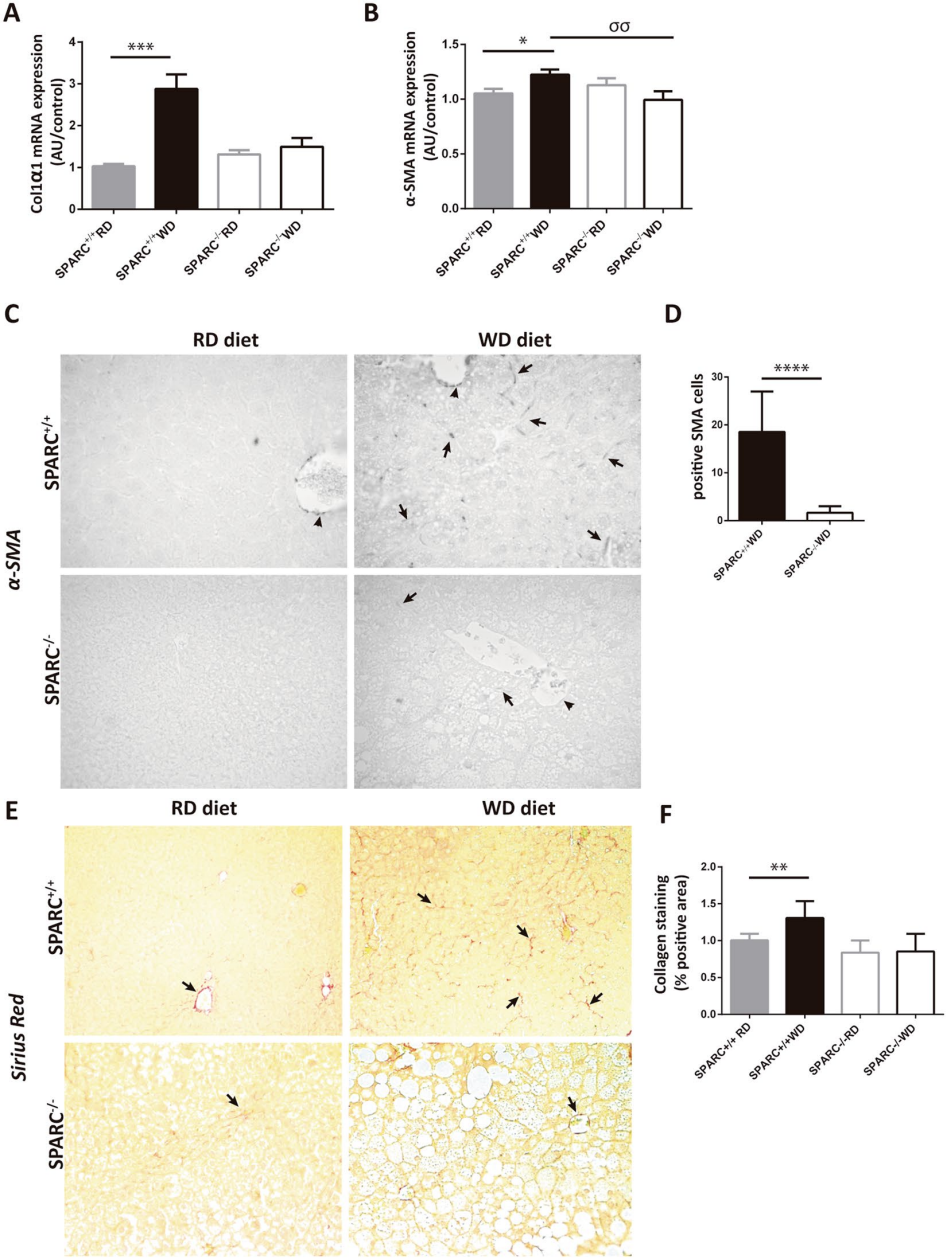
Fibrosis markers decrease in SPARC^−/−^ mice fed with WD diet. (**A**,**B**) Hepatic expression of collagen alpha I, and α-SMA in response to WD feeding compared with controls. *p < 0.05, ***p < 0.001; ^σσ^p < 0.01; Kruskal-Wallis test with Dunn’s post test. n = 6–8 mice per group. (**C**) Immunohistochemistry for hepatic α-SMA. Arrowheads indicate the internal positive control-stained area (portal myofibroblast). Arrows indicate α-SMA positive cells in the perisinusoidal zone. (**D**) α-SMA positive cells quantification. n = 6 mice per group. Twenty random fields were analyzed for each group (x400 magnification) ***p < 0.001, Mann-Whitney U test. (**E**) Representative images of Sirius red staining of liver sections from SPARC^+/+^ and SPARC^−/−^ mice after feeding WD diet. Arrows indicate collagen deposition. Magnification: 200 × B. (**F**) Quantification of Sirius red-positive areas using image analysis. n = 6–8 mice per group. **p < 0.01, Kruskal-Wallis test with Dunn’s post test.

We aimed to check the effects of WD on fibrogenesis in SPARC^−/−^ and SPARC^+/+^ mice. We first analyzed the expression of collagen α I, the most abundant extracellular matrix protein in fibrotic hepatic tissues, and observed that its levels were significantly higher in WD fed SPARC^+/+^ mice compared with SPARC^−/−^ mice (Fig. 6A). Expression of α-SMA, a known marker of HSC activation, was significantly higher in SPARC^+/+^ mice fed with WD (Fig. 6B), which was confirmed by a decrease in α-SMA positive cells in livers form SPARC deficient mice (Fig. 6C,D). In line with these observations, Sirius red staining revealed more perisinusoidal collagen deposition in the liver of SPARC^+/+^ mice compared with SPARC^−/−^ mice (Fig. 6E,F). These results show, as observed in other fibrogenic models, that the absence of SPARC is associated with reduced hepatic fibrogenesis in a NAFLD setting.

## Discussion

The clinical outcome of patients with NAFLD is variable due to host, genetic and environmental factors^33^. Some patients develop mild hepatic injury that rarely progresses to end-stage hepatopathy^34^; however, some of the patients develop cirrhosis and/or hepatocellular carcinoma, even without established cirrhosis^35^. Moreover, the strongest currently known predictor for health outcomes in NAFLD is the severity of fibrosis^36,37^. Nevertheless, the factors driving the progression from simple steatosis to NASH and in particular into fibrosis are poorly defined^38^. In the present work, we provide new data about the role of SPARC during chronic liver injury in NAFLD/NASH. NAFLD patients with elevated SPARC expression exhibited higher markers of apoptosis and profibrogenic gene expression. Results from mice with chronically injured fatty livers showed that SPARC was induced and its expression was associated with liver damage and fibrosis. Moreover, SPARC knockout mice were protected from liver injury and showed less inflammation and fibrosis in a dietary model of NASH. These results also confirm previous studies in mice, which showed that SPARC has a role in liver fibrogenesis^14,39^.

SPARC expression is induced during liver fibrogenesis in different species. In mice, SPARC upregulation was reported in a schistosomiasis model of liver fibrosis^10^, and by our group in chronic thioacetamide intoxication and bile duct ligation. In addition, we showed previously that SPARC, expressed by HSC and LSEC, plays a role in key pathogenic events related to the fibrogenic process such as necrosis of hepatocytes, inflammation, recruitment/activation of HSC as well as in the induction of TGF-β1^14,39^. Little information is available on the role of SPARC in human liver fibrosis, although we and others have shown that SPARC is overexpressed in the liver of cirrhotic patients^11,14^.

Since fibrosis seems to be a major determinant for overall outcome in NAFLD, it is crucial to identify predictors, which patient will progress from steatosis to a fibrogenic state. Classic routine liver injury markers have limited informative value for this setting^7,40,41^. Previous studies have suggested CK18 serum levels (M30), a surrogate marker for epithelial cell apoptosis, as the most consistent single blood marker for diagnosis of NASH^42,43^. In the present study, serum M30 concentrations were elevated in patients with high SPARC mRNA expression in the liver compared to those with low SPARC expression. Although classic liver serum parameters were within normal ranges, high SPARC expression in the liver was associated with nominally higher serum liver parameters^40,44^. This would suggest a stronger NAFLD-associated liver damage in patients with high SPARC. Recently it has been suggested that also necroptosis, a different type of programmed cell death, may be involved in fibrogenesis in NASH^45–47^. The role of necroptosis in NASH patients is still a matter of debate although Gautheron *et al*. observed significant expression of RIPK3 in livers of patients with NASH^28,48^. We found that patients with high SPARC expression exhibited slightly increased RIPK3 mRNA and that expressions of both factors were correlated. Moreover, in a dietary model of NASH using SPARC loss of function mice, higher RIPK3 mRNA levels were detected in the liver. This could indicate a role for SPARC in the development of fibrosis by interaction with necroptosis-associated gene expression in NAFLD.

In the present study, we also found direct associations of genes involved in fibrogenesis and SPARC. Patients with high levels of SPARC had increased hepatic mRNA levels of Col1α1 and TGFβ1. Expressions of both genes were correlated to SPARC mRNA expression. These results are in agreement with more severely injured livers and suggest a stronger fibrogenesis in association with higher SPARC expression. Though, no differences were observed for actual fibrosis score or collagen deposition. In addition, the absence of SPARC was associated with reduced HSC activation as well as proinflammatory and profibrogenic gene expressions in the murine NAFLD model. SPARC mRNA expressions in liver and in adipose tissue were also correlated, suggesting a parallel mechanism or interaction between the two sites. If SPARC expression in adipose tissue is connected to adipose tissue inflammation and fibrosis needs to be determined in future studies.

A key factor in triggering cell death in the liver is the stimulation of the immune system^49^; and chronic inflammation is a potent promoter of fibrogenesis in NASH. Kupffer cells have been known to have a key role in triggering fibrosis via monocyte recruitment and stimulation of hepatic stellate cells; although macrophages can also have a restorative function from injury^50^. One of the most important chemokines involved in macrophage chemotaxis is IP10, also named CXCL10^51^. Both hepatic macrophage infiltration (assessed by the presence of the activation maker F4/80) and IP-10 mRNA expression were reduced in SPARC^−/−^ mice in comparison with wt mice. In line with this, the expression of IL-6, TNF-α, and FAS/CD95 were significantly reduced in livers from SPARC deficient mice. Overall inflammatory cells and chemokines were markedly reduced in SPARC^−/−^ mice, suggesting that decreased fibrosis and the protective effect observed in animals lacking SPARC was in part mediated by a reduced pro-inflammatory hepatic microenvironment. We have also described a potent anti-fibrotic effect in two models of chronic hepatic injury (thioacetamide and bile duct ligation) generated in SPARC^−/−^ mice^14^. Of note, SPARC-deficient mice showed a significant increase of fatty accumulation in the liver upon injury. However, we did not find a correlation between steatosis and the degree of necroinflammation. On the contrary, SPARC^−/−^ mice were protected from liver damage. These data suggest that SPARC^−/−^ mice are protected from hepatic injury induced by fatty acids accumulation and subsequent generation of apoptosis/necrosis, inflammation and fibrosis. The antifibrogenic effect of SPARC inhibition is not restricted to NAFLD and was also observed in other models of chronic liver injury such as TAA and BDL^13^.

In all, our data support our hypothesis that SPARC is increased in NAFLD patients who are at high risk for fibrosis development, and justify further investigations to define the mechanisms behind this association. SPARC could be a possible target to prevent early NAFLD progression to fibrosis.

## Electronic supplementary material

Supplementary Table and Figure
