# Supplementary material for: SPARC expression is associated with hepatic injury in rodents and humans with non-alcoholic fatty liver disease
Source: Sci Rep. 2018 Jan 15;8:725. doi: 10.1038/s41598-017-18981-9 (PMC5768809; doi:10.1038/s41598-017-18981-9)
Supplement: Supplementary file 1 — Supplementary Table and Figure [file 41598_2017_18981_MOESM1_ESM.pdf]

SPARC expression is associated with hepatic injury in rodents and humans with non-alcoholic fatty liver disease

Guillermo Mazzolini, Catalina Atorrasagasti, Agostina Onorato, Estanislao Peixoto, Martin Schlattjan, Jan-Peter Sowa, Svenja Sydor, Guido Gerken, Ali Canbay

[1]Gene Therapy Laboratory, Instituto de Investigaciones Medicas Aplicadas, Universidad Austral, Buenos Aires, Argentina. [2]CONICET (Consejo Nacional de Investigaciones Científicas y Técnicas), Buenos Aires, Argentina. [3]Department for Gastroenterology and Hepatology, University Hospital, University Duisburg-Essen, Essen, Germany. [4]Department of Gastroenterology, Hepatology and Infectious Diseases, Otto-von-Guericke University, Magdeburg, Germany
[#]equal contribution

Supplementary Information

Supplementary table 1: Oligonucleotide sequences used for qrt-PCR.

Human			
NCBI symbol	***Gene***	***Forward (5´3´)***	***Reverse (5´3´)***
**sparc**	sparc	AAACCGAAGAGGAGGTGGTG	GCAAAGAAGTGGCAGGAAGA
**Fabp1**	L-fabp	GCAGAGCCAGGAAAACTTTG	CACCCCCTTGATATCCTTCC
**Fatp5**	Fatp5	GCCCTGCCCTCTTCATCTAT	CCCCAGATAAGGACAGCATC
**Acta2**	αSma	TTCGTTACTACTGCTGAGCGTGAG	AAGGATGGCTGGAACAGGGTC
**Tgfb1**	Tgfb1	GTACCTGAACCCGTGTTGCT	GAACCCGTTGATGTCCACTT
**Tnf**	Tnfα	GCCCCCAGAGGGAAGAGTTCCC	GAGCTCCACGCCATTGGCCA
**Ripk3**	Ripk3	TGGCCCCAGAACTGTTTGTT	GGATCCCGAAGCTGTAGACG
**Hprt**	Hprt	GACCAGTCAACAGGGGACAT	CTTGCGACCTTGACCATCTT

Mouse			
NCBI symbol	***Gene***	***Forward (5´3´)***	***Reverse (5´3´)***
**sparc**	sparc	CCACACGTTTCTTTGAGACC	GATGTCCTGCTCCTTGATGC
**Fasn**	Fas	CAAGGAGGCCCATTTTGCTG	CAGGTTGGTGTACCCCCATT
**IP-10**	Cxcl10	GGTTGCCAAGCCTTATCGGA	ACCTGCTCCACTGCCTTGCT
**Acta2**	αSma	TTCGTTACTACTGCTGAGCGTGAG	AAGGATGGCTGGAACAGGGTC
**Tnf**	Tnfα	ACCGTGACAATCCCCTGTAA	AGGGACGCACTCACTTTCTC
**Il6**	IL-6	AGTTGCCTTCTTGGGACTGA	TCCACGATTTCCCAGAGAAC
**Col1a1**	Col1α1	CCTACATGGACCAGCAGACTG	GGAGGTCTTGGTGGTTTTGTA
**Gapdh**	Gapdh	GGGGCTGCCCAGAACATCAT	GCCTGCTTCACCACCTTCTTG

Whether FFA induce SPARC expression in an *in vitro* model, primary human hepatocytes were treated with FFA at a concentration of 0.5 and 1 mM. As shown in supplementary figure 1, mRNA expression of SPARC was significantly increased in treated samples compared with controls (at 0.5 mM). In addition, incubation of hepatocytes with FFA induces the expression of TNF-α, RIP3k, TGF-β1, collagen-1α, FASN, and FABP-1 mainly at 1mM. Although SPARC is induced after FFA incubation at 0.5 mM no changes were observed for TNF-α, RIP3k, TGF-β1, collagen-1α, FASN at the same concentration.

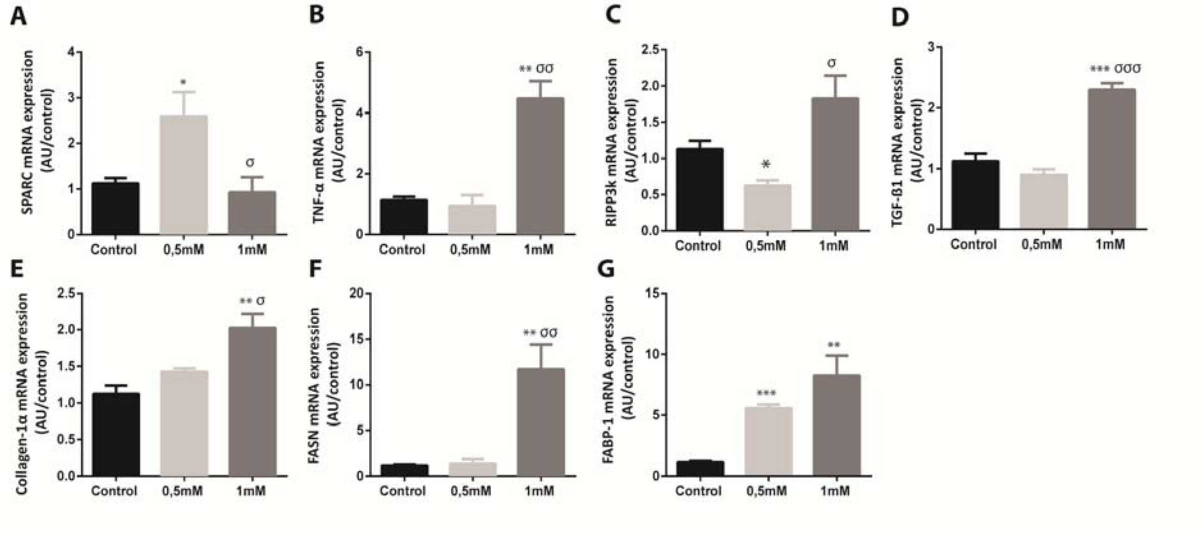

Supplementary figure 1: In vitro treatment with FFA enhances SPARC mRNA expression of hepatocytes. SPARC (A) and TNF-α, RIP3k, TGF-β1, collagen, and the fatty acid metabolism genes FASN and FABP-1 (B-G) expression analysis of primary human hepatocytes culture with 0.5 or 1 mM NEFA. Complementary DNA was synthesized and was subjected to qPCR for the expression the transcripts. *, compared control cells versus treated cells; $^{\sigma}$, 1mM versus 0.5mM treated cells. *p<0.05; **p<0.01, ***p<0.001; $^{\sigma}$ p<0.05; $^{\sigma\sigma}$ p<0.01, $^{\sigma\sigma\sigma}$ p<0.001; Mann-Whitney T test.
